# Identifying Spanish Areas at More Risk of Monthly BTV Transmission with a Basic Reproduction Number Approach

**DOI:** 10.3390/v12101158

**Published:** 2020-10-13

**Authors:** Cecilia Aguilar-Vega, Jaime Bosch, Eduardo Fernández-Carrión, Javier Lucientes, José Manuel Sánchez-Vizcaíno

**Affiliations:** 1 VISAVET Health Surveillance Centre, Animal Health Department, Faculty of Veterinary Medicine, Complutense University of Madrid, 28040 Madrid, Spain; jbosch@ucm.es (J.B.); eduardofc@ucm.es (E.F.-C.); jmvizcaino@ucm.es (J.M.S.-V.); 2 Department of Animal Pathology (Animal Health), AgriFood Institute of Aragón IA2, Faculty of Veterinary Medicine, University of Zaragoza, 50013 Zaragoza, Spain; jlucien@unizar.es

**Keywords:** bluetongue virus, arbovirus, Culicoides, blood-feeding vector, epidemiology, modeling, risk analysis, livestock, basic reproduction number, machine learning

## Abstract

Bluetongue virus (BTV) causes a disease that is endemic in Spain and its two major biological vector species, *C. imicola* and the Obsoletus complex species, differ greatly in their ecology and distribution. Understanding the seasonality of BTV transmission in risk areas is key to improving surveillance and control programs, as well as to better understand the pathogen transmission networks between wildlife and livestock. Here, monthly risk transmission maps were generated using risk categories based on well-known BTV *R*_0_ equations and predicted abundances of the two most relevant vectors in Spain. Previously, *Culicoides* spp. predicted abundances in mainland Spain and the Balearic Islands were obtained using remote sensing data and random forest machine learning algorithm. Risk transmission maps were externally assessed with the estimated date of infection of BTV-1 and BTV-4 historical outbreaks. Our results highlight the differences in risk transmission during April-October, June-August being the period with higher *R*_0_ values. Likewise, a natural barrier has been identified between northern and central-southern areas at risk that may hamper BTV spread between them. Our results can be relevant to implement risk-based interventions for the prevention, control and surveillance of BTV and other diseases shared between livestock and wildlife host populations.

## 1. Introduction

Bluetongue (BT) is an infectious and arboviral disease with a worldwide distribution, that affects primarily ruminants [1]. Bluetongue is caused by bluetongue virus (BTV), the type species of the *Orbivirus* genus [2]. Numerous serotypes of BTV have been identified, particularly in recent years due to the intensification of BTV sequencing [3]. Clinical forms of BT vary greatly depending on the affected species, sheep and white-tailed deer being the most susceptible ones to the disease [1]; however, the specific BTV strain is also important, as shown by the greater susceptibility of cattle during the BTV-8 circulation in north and central Europe [4]. Due to its transboundary dissemination and the economic losses that BT produces, it is a listed disease of the World Organization for Animal Health (OIE) [5].

Five different BTV serotypes have historically affected Spain (BTV-1, 2, 4, 8 and 10), differing in their spatial distribution. BTV-2 circulated in Minorca and eastern Majorca in 2000 and was deemed to have been eradicated in 2003 [6]. BTV-10 (1956–1960) was mainly recorded in southwestern and central-western areas of mainland Spain [7]. BTV-4 had a similar distribution, although it expanded more in southern areas of mainland Spain, and outbreaks were also notified in 2003 in west Minorca. BTV-1 spread across a wider geographical area causing outbreaks in northern livestock holdings during 2007–2009 [7,8], and having an overall similar distribution to BTV-4 in southwestern and central-western areas [9]. On the contrary, there were sporadic BTV-8 notifications in livestock holdings from 2008 to 2010 in both north and south areas of mainland Spain [7].

*Culicoides* spp. biting midges serve as biological vectors for the typical serotypes of BTV (BTV-1-24) [1,10], although secondary transmission routes have been identified, namely, transplacental [11,12], iatrogenic [13], oral transmission [12], and even a role for other vectors has been suggested [14,15,16,17]. The Obsoletus complex species are considered major vectors of BTV in central and northern European regions [17,18], while *C. imicola* is the major vector in the Mediterranean Basin [10,18]. Although, other *Culicoides* species have been identified as potentially competent vectors [18,19,20], in the present work we will be focusing exclusively on *C. imicola* and the Obsoletus complex species, since they are considered to be the most important vectors of BTV in Spain [21,22].

Since BTV is an arthropod-borne virus (arbovirus), the distribution of BTV is directly related to the distribution of its vector [10]. *Culicoides* species distribution is related to different climatological, topographic and anthropogenic factors [22,23,24,25,26]. BTV transmission is highly influenced by temperature. It influences the vector lifespan and metabolism, as well as the period between the vector ingestion of the virus in the blood meal and the virus infection of the salivary glands—enabling virus transmission—known as the extrinsic infection period (EIP) [27]. The basic reproduction number (*R*_0_) is defined as “the expected number of secondary cases following the introduction of one infectious individual into a fully susceptible population” [28]. Derived from this definition, when *R*_0_ > 1 the virus will spread among the susceptible host population, but when *R*_0_ < 1 the virus will eventually disappear [28]. However, due to the assumptions and uncertainties that affect *R*_0_ studies, this threshold (*R*_0_ > 1 or *R*_0_ < 1) is not always met [29]. In addition, *R*_0_ studies of vector-borne viruses are more complex, since they have to take into consideration the biology of its vector; and they are even more so when various hosts and vectors are involved [30,31]. Risk maps based on *R*_0_ can show areas at more risk of virus transmission, and therefore, they could be useful for decision making [32]. This kind of map has been previously used for different vector-borne diseases, such as West Nile [33], Lyme disease [34], Zika [35]; and for BT in Europe [36], Switzerland [37], the Netherlands [32] and Austria [38].

Taking into account the seasonality of BTV is key to better understand the disease dynamics and the modification of the risk of transmission during different times of the year [38,39]. In this study, we have assessed BTV seasonality in Spain based on the *R*_0_, taking into account the major European vectors: *C. imicola* and the Obsoletus complex species. 1-km^2^ monthly *Culicoides* spp. abundance maps for mainland Spain and the Balearic Islands (from April to October) have been generated using random forest algorithm and satellite-derived variables that are known to influence *Culicoides* spp. distribution.

## 2. Materials and Methods

### 2.1. Monthly Culicoides spp. Maps and Environmental Variables

Data of *Culicoides* spp. catches in Spain were provided by the Spanish Bluetongue National Surveillance Program. The entomological surveillance of this program was initiated in 2004, and is still ongoing to this date [40]. Briefly, CDC UV-light traps are placed near livestock farms weekly from dusk until dawn [41]. *C. imicola* and the Obsoletus complex species (*Culicoides obsoletus* and *Culicoides scoticus*) are the major vector species for BTV in Spain [21,22], therefore, we used data of these biological vectors from 2005 until 2015 to generate monthly abundance maps from April to October. This period coincides with the peak abundances of these species and with the greater transmission of BTV in Spain [21,41], as for northern countries such as the Netherlands [32]. To ensure the homogeneity and representativeness of the subsamples, we removed from the study data from traps that could have a bias. Hence, we exclusively selected traps with at least one catch per month during the seven months included in the study, also ensuring a maximal geographical coverage. From those, when the proximity was less than 10 km, one of the sampling sites was randomly removed to avoid spatial autocorrelation [26]. We finally used 331 sampling sites per month and per species. We chose the maximum abundance per site for *C. imicola* and the Obsoletus complex species, to mitigate the possible abundance reduction due to climatological or technical problems, as an approximation of the real monthly *Culicoides* spp. abundance [42].

A review of scientific literature was conducted to choose significant variables to generate predictive models for the Spanish principal vectors [22,23,24,25,42,43,44,45,46,47]. The selected variables can be classified into six different categories: climatic, vegetation indices, host, orography, land cover and soil composition.

Within climatic variables, Land Surface Temperature (LST) of day-time (LSTd) and night-time (LSTn) were retrieved from National Aeronautics and Space Administration’s (NASA) Moderate Resolution Imaging Spectroradiometer (MODIS) product MOD11A2 [48] as an accurate estimate of the day-time and night-time temperature for each day in the study period [49] and this was downloaded from the Level-1 and Atmosphere Archive & Distribution System (LAADS) Distributed Active Archive Center (DAAC) (https://ladsweb.nascom.nasa.gov/). Monthly precipitation and wind speed (m/s) at 10 m above ground level were retrieved from the approximately 1-km^2^ spatial resolution WorldClim dataset [50].

Vegetation indices are widely used to model arthropod species, being the Normalized Difference Vegetation Index (NDVI) the most common in predictive models due to its utility [51]. NDVI alongside the Enhanced Vegetation Index (EVI) and the medium-infrared (MIR) reflectance band were retrieved from MODIS product MOD13Q1 [52] and downloaded from the LAADS DAAC. Variables derived for MODIS products were acquired from 2005-2015, and mean maps were generated for each month included in the study.

Host availability has been proven to be useful for *Culicoides* spp. distribution modeling [53,54]. From the Gridded Livestock of the World v2.0 [55] of approximately 1-km grid cell size, cattle, sheep and goat densities were retrieved and unified as a unique variable: livestock density [26]. The estimation of presence/absence of red deer was obtained from the proportion of suitable red deer habitat at approximately 1-km^2^ spatial resolution [56].

Altitude values were obtained from the Global 30 Arc-Second Elevation (GTOPO30) provided by the U.S. Geological Survey [57]. Land cover and soil type can limit *Culicoides* spp. breeding sites [58]. Land cover was retrieved from the annual 300 meters’ resolution Climate Change Initiative (CCI) Land Cover provided by the European Space Agency [59]. This dataset was reduced to seven variables according to the midge habitat suitability [47,60]: rainfed cropland (LC1), irrigated cropland (LC2), mix of cropland and natural vegetation (LC3), broadleaved tree cover (LC4), mix of tree/shrub cover and grassland (LC5), grassland (LC6) and urban areas (LC7). Using ArcMap^TM^ v10.4.1. (Esri^®^), we created a 1-km^2^ grid of the extension of the study and obtained the percentage of each land cover type. The percentage of clay, sand, silt and the topsoil organic carbon content were obtained from the dataset ‘Topsoil physical properties for Europe’ [61] and “OCTOP: Topsoil Organic Carbon Content for Europe” [62], respectively; available at the European Soil Data Centre (ESDAC; esdac.jrc.ec.europa.eu).

All the predictor rasters were transformed into a 1-km^2^ spatial and monthly resolution, and to the same extension and projection in ArcMap^TM^.

For each model, we randomly generated a training (70%) and test (30%) dataset [23]. The latter was used to assess model performance. We chose random forest (RF) to develop our models since it performed better than other machine learning algorithms for *Culicoides* spp. distribution models in several studies [26,45]. RF grows a specified number of decision trees from randomly bootstrapped samples, and their results are finally aggregated to give a prediction [63,64]. At each node in the splitting process, the variable that reduces the node impurities from a random subsample of them is chosen [63]. When all the trees are built, the average of the decreases provides the variable importance measure for each variable through the mean decrease Gini in classification and the increase in node purities for regression problems [64]. In this work, we used the above-mentioned measures for variable importance measurement. We chose the number of variables randomly selected at each node split which reduces the final mean square error (MSE) of the model. In addition, RF is useful for datasets with highly correlated predictors, being the most relevant ones selected as the most important variables [65].

We first generated monthly probability of presence maps, henceforth occurrence maps, for *C. imicola* and the Obsoletus complex species, to include those as a variable for abundance maps [24,26]. For occurrence maps, *Culicoides* spp. catch data was transformed into presence/absence classes, and the 21 variables listed above were used for modeling. In the case of imbalanced datasets, we used the Synthetic Minority Over-sampling Technique (SMOTE) algorithm, to oversample the minority class and randomly under-sample the majority class [66]. Models’ performance were assessed in terms of sensitivity (proportion of real positives classified as such), specificity or recall (proportion of real negatives classified as such), precision (proportion of true positives among predicted positives), the area under the receiver operating characteristic curve (AUC) and the F1 score. All these measures range from 0–1. AUC measures the discriminative power of the model between presence/absence classes. An AUC of 0.5 means that the model is not better than a random guess; values from 0.5–0.7 correspond to poor discrimination, 0.7–0.8 acceptable, 0.8–0.9 excellent, and 0.9–1 exceptional discrimination power [67]. The F1 score is calculated as the harmonic average of precision and recall, and it is a summary of the precision-recall curve [68]. The precision-recall curve best measures the performance of the model when the data set is imbalanced, with the majority class being negative [69]. The value of the F1 score means that the model is not better than random guess varies, and a value of 1 is the maximal model performance. For abundance models, we transformed the absolute catches of *Culicoides* spp. to *log*_10_(C+1), C being the number of individuals caught at each site. We evaluated the performance of abundance models using the mean absolute error (MAE) and root mean squared error (RMSE).

All the models were generated in R v.3.6.3 [70] using the following packages: “randomForest” [64], “DMwR” [71], “caret” [72], “raster” [73], and “pROC” [74].

### 2.2. Transmission Risk Maps Based on the Basic Reproduction Number (R_0_)

From the different *R*_0_ equations proposed in the literature for BTV [31,32,37,75], we chose the two-vector approach taken by Turner et al. [31] that was adapted from the one proposed by Gubbins et al. [75], since the major BTV vectors in Spain are quite different in terms of distribution and ecology [21], as well as vector capacity [76]. In Turner et al. [31], they only considered in their formulation cattle and sheep as the two hosts; however, we have combined small ruminants (sheep and goat) as one host since in Spain the number of goats is considerably high (more than 2.5 million in mainland Spain and the Balearic Islands [77]). We have considered all susceptible hosts as susceptible, not infectious or recovered, because our goal was to highlight areas at risk of BTV transmission. Moreover, more than two vector species are considered here because the Obsoletus complex is formed by *C. obsoletus* and *C. scoticus*.

We have maintained the same notation as Turner et al. [31] for the *R*_0_ equation (Equations (1) and (2)), of which the parameters are gathered in Table 1.

(1) $$R_{0}=\sqrt{\frac{1}{2}\left[\left(R_{11}{\;+\;R}_{22}\right)\;+\;\sqrt{{\left(R_{11}{\;+\;R}_{22}\right)}^{2}\;-\;4\left(R_{11}R_{22}\;-{\;R}_{12}R_{21}\right)}\right]},$$

(2) $$R_{11}=\left(\frac{b_{1}\beta_{1}a_{1}^{2}}{{µ}_{1}}\right)\left(\frac{v_{1}}{v_{1}{\;+\;µ}_{1}}\right)\left(\frac{\varphi_{1}^{2}m_{C1}}{r_{C}{\;+\;d}_{C}}\;+\;\frac{{\left(1-\varphi_{1}\right)}^{2}m_{S1}}{r_{S}{\;+\;d}_{S}}\right)\;R_{22}=\left(\frac{b_{2}\beta_{2}a_{2}^{2}}{{µ}_{2}}\right)\left(\frac{v_{2}}{v_{2}{\;+\;µ}_{2}}\right)\left(\frac{\varphi_{2}^{2}m_{C2}}{r_{C}{\;+\;d}_{C}}\;+\;\frac{{\left(1-\varphi_{2}\right)}^{2}m_{S2}}{r_{S}{\;+\;d}_{S}}\right)\;R_{12}=\left(\frac{b_{2}\beta_{1}a_{2}a_{1}}{{µ}_{2}}\right)\left(\frac{v_{2}}{v_{2}{\;+\;µ}_{2}}\right)\left(\frac{\varphi_{2}\varphi_{1}m_{C1}}{r_{C}{\;+\;d}_{C}}\;+\;\frac{\left(1-\varphi_{2}\right)\left(1-\varphi_{1}\right)m_{S1}}{r_{S}{\;+\;d}_{S}}\right)\;R_{21}=\left(\frac{b_{1}\beta_{2}a_{1}a_{2}}{{µ}_{1}}\right)\left(\frac{v_{1}}{v_{1}{\;+\;µ}_{1}}\right)\left(\frac{\varphi_{1}\varphi_{2}m_{C2}}{r_{C}{\;+\;d}_{C}}\;+\;\frac{\left(1-\varphi_{1}\right)\left(1-\varphi_{2}\right)m_{S2}}{r_{S}{\;+\;d}_{S}}\right)$$

where subscript 1 denotes *C. imicola*, 2 the Obsoletus complex species, *C* cattle, and *S* small ruminants; and *b* being the probability of transmission from vector to host, *β_j_* the probability of transmission from a host to vector type *j*, *a_j_* the biting rate for vector type *j*, *µ_j_* the natural mortality rate of the vector, *v_j_* the virogenesis rate in the vector, *φ _j_* the proportion of vectors type *j* attracted to cattle, *m_ij_* the ratio of vector type *j* to hosts type *i*, *r_i_* the recovery rate of host type *i*, and *d_i_* the mortality rate of host type *i*. Both vectors have only differed in terms of *m_ij_* and *a_j_*.

*φ_j_* depends on vector preference for cattle or small ruminants (Table 1). Both *C. imicola* and Obsoletus complex species prefer feeding on cattle rather than small ruminants [88]. We applied an antilog transformation to the predictive abundance of *Culicoides* spp. to calculate *m_ij_*. Although several studies have pointed out the lack of correlation between UV-light trap catches and biting events [89,90,91,92,93], we have assumed that UV-light traps act in a similar way to a host and that the predicted abundance estimated here is an approximation of *m_ij_* [36]. We used host data (the Gridded Livestock of the World v2.0 [55], merging sheep and goat to obtain small ruminants data), to estimate a minimum threshold of 10 animals [32], below which they would not contribute significantly to BTV transmission. Then, we transformed into integers rasters of vectors and hosts. In addition, *m_ij_* could only range between 0–5000 [75].

Some parameters are well known to be temperature-dependent (*a_j_*, *v_j_* and *µ_j_*) [84,94]. At higher temperatures biting, virogenesis rates and lifespan are shortened [85,94]. Replication of BTV in *Culicoides* spp. has not been found with temperatures <15 °C [94], and at approximately that temperature the midge lifespan is higher but decreases non-linearly alongside temperature [87]. Therefore, to make monthly *R*_0_ prediction maps we required monthly ground-level temperature for mainland Spain and the Balearic Islands that we obtained from the WorldClim dataset [50]. For the estimation of *µ_j_* we used the formulation proposed by Fernández-Carrión et al. [87], in which *µ*_j_ is calculated by estimating intermediate values from [85,86] using the Hermite cubic interpolation in Matlab [95]. In addition, we have established that BTV transmission is very unlikely to occur below 13 °C [38,94]. We used the monthly mean maximal temperature from the WorldClim dataset [50] to apply this threshold. *v_j_* was set to zero when the temperature is less than 10.4071 °C.

We have implemented the *R*_0_ equations in R [70], and managed maps using the “raster” package [73]. To determine the degree of contribution of each vector species, we obtained the *R*_0_ for each vector using the one vector formulation ($\sqrt{R_{11}}$ and $\sqrt{R_{22}}$) [75]. We defined three risk categories for each monthly map according to the natural break classification criteria [96] in ArcMap^TM^.

To externally assess the performance and usefulness of the monthly transmission risk maps we used historical data from 2007–2018 of BTV-1 (11,482) and BTV-4 (506) outbreaks from the European Animal Disease Notification System (ADNS) database [97], excluding BTV outbreaks in wildlife. We used an estimation of 14 day-lapse between the day of infection and the date of suspicion [98]. The suspicion date was calculated when missing from the mean lapse time between the suspicion and confirmation dates from observations with data (approximately 20 days). We calculated the percentage of outbreaks that fall into the three different risk categories. We compared the observed BTV outbreaks in each risk category with the number of outbreaks expected based on the expected probability, which is derived from the proportion of cells of each category in every monthly risk map. We used the Χ^2^-square goodness of fit test in R [70] only when the number of observed outbreaks was higher than 100.

## 3. Results

### 3.1. Monthly Culicoides spp. Maps

**Figure 1:**
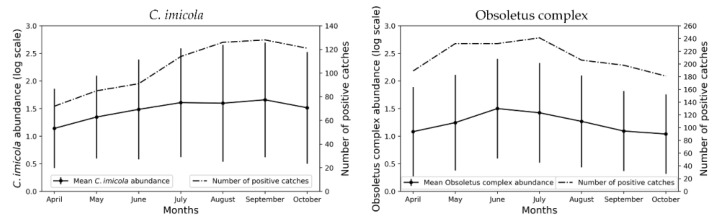
Mean abundance of *Culicoides* spp. in positive sampling sites and the number of positive catches for the 331 sampling site observations for each month of the study period. The abundance of *Culicoides* spp. was transformed to *log*_10_(*C*+1), *C* being the number of *Culicoides* spp. Vertical lines show the standard deviation of the mean for each month.

**Figure 2:**
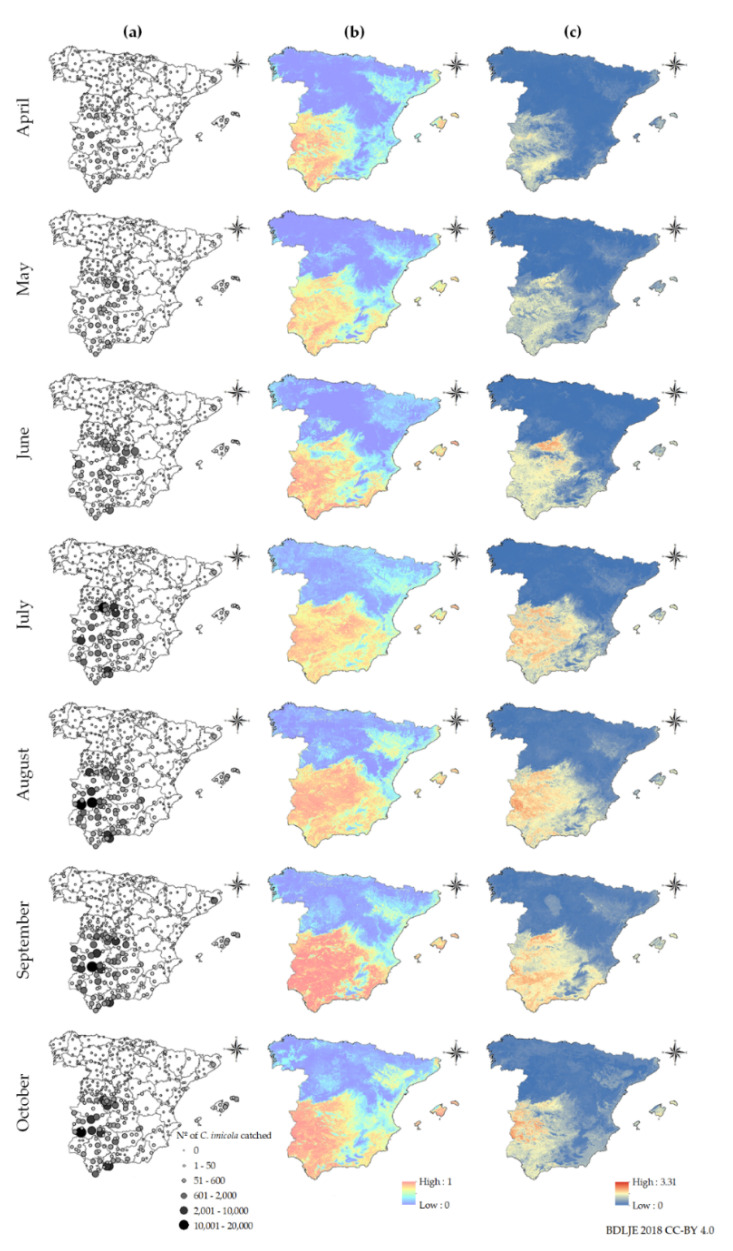
Monthly *C. imicola*’s: (**a**) abundance in sampling sites, (**b**) occurrence model, (**c**) abundance model. Administrative boundaries provided by Instituto Geográfico Nacional (ign.es); BDDAE CC-BY 4.0.

**Figure 3:**
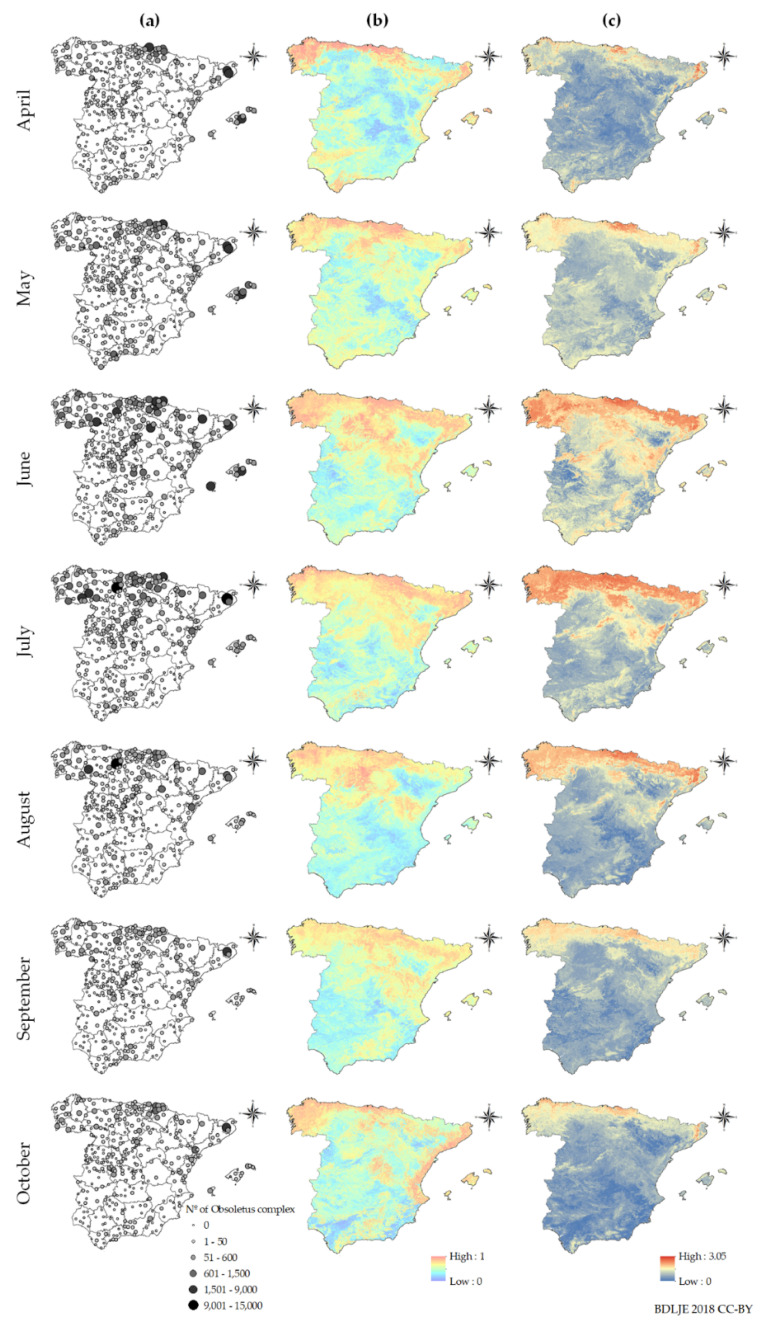
Monthly Obsoletus complex’s: (**a**) abundance in sampling sites, (**b**) occurrence model, (**c**) abundance model. Administrative boundaries provided by Instituto Geográfico Nacional (ign.es); BDDAE CC-BY 4.0.

The peak of maximal positive catches and average abundance for *C. imicola* was September, while for the Obsoletus complex species it was July and June (Figure 1). We have generated monthly occurrence and abundance models for *C. imicola* and the Obsoletus complex species (Figure 2 and Figure 3) from April to October. The seasonality and distribution of these vectors are reflected in the prediction maps. *C. imicola* is restricted to south and southwestern areas of mainland Spain and the Balearic Islands, while the Obsoletus complex species are widespread in all the territory.

**Figure 4:**
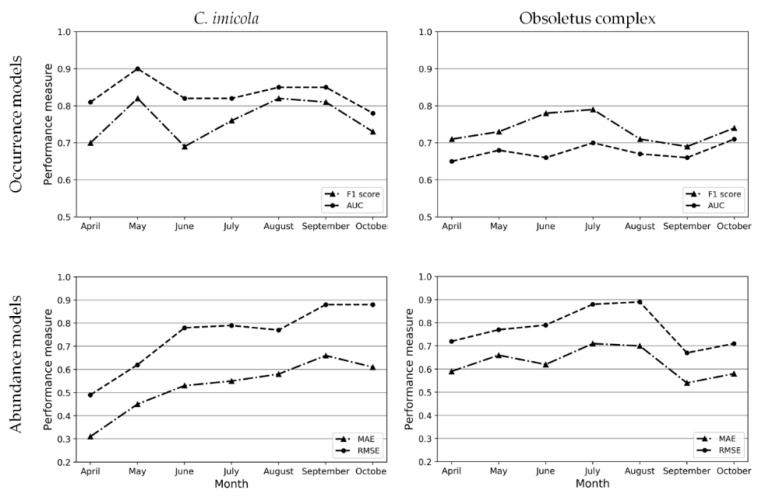
Performance of *Culicoides* spp. occurrence and abundance models. For occurrence models, the F1 score and area under the receiver operating characteristic curve (AUC) are shown, while for abundance models mean absolute error (MAE) and root mean square error (RMSE) are shown.

For occurrence models the majority of the datasets were imbalanced in favor of the negative class in the case for *C. imicola* datasets, and in favor of the positive class for Obsoletus complex species datasets; thus, the SMOTE algorithm was applied (Table S1). The *C. imicola* occurrence models have an average AUC of 0.83 in contrast with the Obsoletus complex species average of 0.68 (Figure 4, Table S2). The *C. imicola* models outperformed the Obsoletus species in terms of sensitivity (average 0.81 vs. 0.70) and specificity (average 0.86 vs. 0.65). Taking into account that the test dataset reflects the class distribution of the original dataset, in the case of imbalanced datasets that favors the negative class (*C. imicola* datasets), specificity is expected to be better. On the other hand, when the majority class is the positive one (as in the Obsoletus complex species datasets), precision is expected to outperform the recall/sensitivity measure, as happened in most of the performances of the Obsoletus complex species occurrence models. Obsoletus complex species models yielded better precision than *C. imicola* models (average 0.78 vs. 0.72). In terms of the F1 score, the difference between the average of both vectors is low: 0.76 for *C. imicola* models and 0.74 for Obsoletus species models. The average performance of the abundance models is quite similar between *C. imicola* and the Obsoletus complex species. The major discrepancy is in the MAE measure, with the *C. imicola* models performing better (0.53 vs. 0.63); followed by RMSE (0.74 vs. 0.78). As expected for the months of maximal abundance for each vector, the error of the correspondent models is higher.

Table S3 gathers the five most important variables for all the *Culicoides* spp. models. Climatic predictors are very relevant for all models, vegetation indices contribute in the majority of monthly models, and livestock density, altitude and soil type for some of them. Land cover predictors and probability of presence of red deer never appeared among the most significant predictors for any of the models.

### 3.2. Transmission Risk Maps

**Figure 5:**
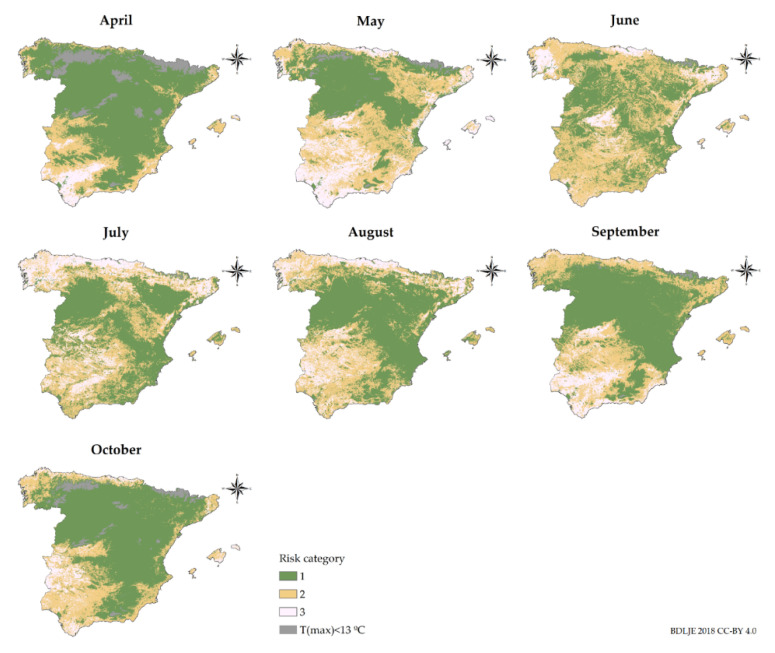
Monthly BTV transmission risk maps in Spain. There are represented three risk categories: low (1), medium (2) and high (3). These categories were defined for each monthly map according to the natural break classification criteria [96] in ArcMap^TM^. Gray areas show areas where the mean maximal temperature is less than 13 °C. Administrative boundaries provided by Instituto Geográfico Nacional (ign.es); BDDAE CC-BY 4.0.

**Figure 6:**
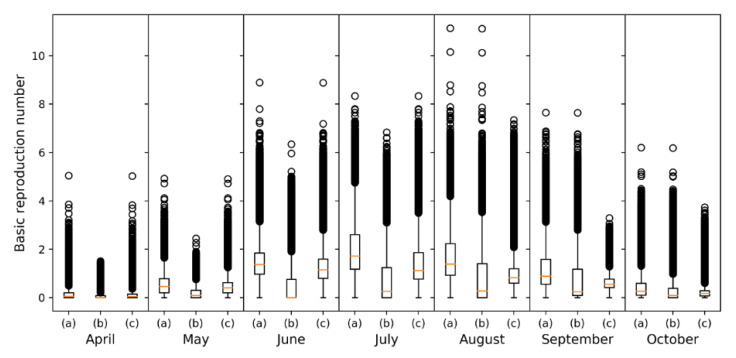
Monthly *R*_0_ values for (**a**) the two-vector formulation, (**b**) one-vector formulation for *C. imicola* and (**c**) one-vector formulation for the Obsoletus complex species.

Monthly risk maps of BTV transmission were generated from April-October as can be seen in Figure 5. A clear seasonality closely associated with vector abundance leads to increased BTV transmission risk during the summer months (Figure 1, Figure 2 and Figure 3). This association was expected since vector abundance highly influences the third component of the *R*_0_ equation, but also the first and second components are driven by temperature-dependent variables (Equation (2)). Looking at the monthly results, in April, mean *R*_0_ was low (0.15), but it increased in May (0.53) and June (1.45), until it reached a peak in July (1.95). In August, the mean *R*_0_ slightly decreased (1.64); and it decreased more significantly in September (1.14) and October (0.43). *R*_0_ values only considering *C. imicola* as a vector, were higher in July-September, peaking in August; while in the case of the Obsoletus complex species *R*_0_ values were higher during June-August, reaching a peak in July (Figure 6).

Hence, according to our results, June-August are the months of greater transmission in terms of the *R*_0_ since during these months the abundance of the Obsoletus complex species is still high and the abundance of *C. imicola* is rising (Figure 1). The Obsoletus complex species influence the overall *R*_0_ more significantly since it is more widespread, and therefore, contribute more to the increase of the *R*_0_ in the hole territory; while *C. imicola* is restricted to south and central areas (Figure 2 and Figure 3). Proof of this is the gradual increase of infection for BTV-1 since June, when the abundance of the Obsoletus complex species peaks (Figure 1), in contrast with BTV-4 that has only circulated in south and central-western areas of mainland Spain.

As can be seen in Figure 5, in mainland Spain there is a clear low-risk area of separation between medium and high transmission risk areas between north and central-southern at-risk areas. In June this barrier it is not as clear, although medium risk areas are not continuous in that area.

The external assessment of the monthly BTV transmission risk maps shows a good fit of the models for BTV, being better for BTV-4 than BTV-1 (Table 2). The majority of outbreaks fell in areas of high and medium risk: for BTV-1, 97.19% in June, 96.67% in July, 95.26% in August, 87.58% in September and 89.77% in October; whilst for BTV-4, 94.94% in September and 94.12% in October. Only in September and October did more than 10% of BTV-1 outbreaks occur in predicted low transmission risk areas. However, in some of these months BTV transmission, inferred by the estimated date of infection, did not exceeded 10 reports during the 2007–2018 period. Therefore, this data does not allow to draw statistically significant conclusions but was included here due to its informative nature. Supporting the results of the external assessment, we found for each monthly risk map with more than 100 outbreaks for each serotype statistical significant differences between all observed BTV outbreaks and the number of expected BTV outbreaks calculated from the expected probability with the Χ^2^-square goodness of fit test (Table 3). Thus, this result suggests that the observed BTV outbreaks in a risk category reflect the importance of the risk areas rather than the surface size of them.

We have encountered a discrepancy between the highest monthly *R*_0_ values and the peak of outbreak notifications; this leap will be discussed later on.

## 4. Discussion

We have generated monthly BTV transmission risk maps based on the two-host, two-vector *R*_0_ formulation [31]. Few BTV *R*_0_ studies have been conducted in Spain [79,98], and none have estimated the *R*_0_ for all mainland Spain and the Balearic Islands nor have they considered both BTV vectors separately. Being able to estimate the *R*_0_ in the hole territory is necessary to better understand the transmission risk in a country, in comparison to the estimation of *R*_0_ only on *Culicoides* spp. sampled areas [32]. Hence, modeling the distribution of the main vector species of *Culicoides* is essential. Machine learning algorithms are powerful tools for this task, especially RF [26,45,99]. However, knowing the intrinsic characteristics of the datasets we are working with is essential to correctly apply and interpret the performance of the resulting models. The use of the AUC in this kind of imbalanced dataset is misleading and can lead to wrong conclusions, proving that AUC is not always the most convenient parameter to assess the performance of classification models [69]. Thus, although the *C. imicola* occurrence models outperformed the Obsoletus complex species models (Figure 4, Table S2), the extent of such outperformance cannot be easily quantified since in both cases the original datasets were imbalanced, but with different classes. Our monthly *Culicoides* spp. models (Figure 1, Figure 2 and Figure 3) are in agreement with the vector seasonality historically recorded for Spain [21], proving the suitability of the subsampled sampling sites chosen for modeling. Despite entomological surveillance programs not being designed specifically for the generation of species distribution modeling, predictive abundance models derived from them have proven to be effective and useful for the generation of disease models [26]. In addition, the external assessment of the monthly models shows their great performance (Table 2), i.e., for the month of the highest number of outbreaks according to the estimated date of infection, 89.77% for BTV-1 and 94.12% for BTV-4 fell in areas of high and medium risk of transmission. In the external assessment, in some months the medium risk category areas had a higher proportion of outbreaks than the high risk category (Table 3). This is explained by the classification method used here (natural breaks [96]) that does not always divide the data into balanced categories. The medium and high risk categories for April, September and October maps account for less than 50% of the total extension of the map, and in every monthly map the high risk area is smaller than the medium risk area.

The two-host, two-vector approach is essential for a country such as Spain in which major vectors are so different in terms of ecology and distribution [21]. These differences could hardly be reflected in the model due to the existing gaps in knowledge in some parameters that are especially relevant for *R*_0_ studies, namely, *β_j_*, *a_j_*, *v_j_* and *µ_j_*. All of these are derived from studies performed in *Culicoides sonorensis*, the principal BTV American vector [1,10], although some have been adapted to *C. imicola* [79,87]. When future studies provide new data for the estimation of these parameters for *C. imicola* and the Obsoletus complex species, they could easily be implemented in our model. We deemed that our approach of estimating *µ_j_* [87] is more precise than the usual formulation of *µ*(*θ*) = 0.009^0.16*θ*^ [75,100], which does not take into account *Culicoides* spp. thermal limits, and therefore is not precise for low and high temperatures. The same issue can be attributed to the estimation of *a_j_* and *v_j_* [84]. This implies a limitation in our study since we have used mean predicted temperatures [50]; and while these could be under the temperature transmission threshold [94], temperature above the mean could have allowed BTV transmission. Sheep and goats have been considered equal in terms of duration of viremia and mortality. This might not be accurate, in particular for the mortality parameter, since goats are usually asymptomatic [1]. In addition, according to several studies that compared *Culicoides* spp. catches with UV-light traps and animal-baited traps, *Culicoides* spp. biting events are not faithfully represented by UV-light trap catches [89,90,91,92,93]. The majority of the literature in this respect agrees that *C. imicola* [90,91] and the Obsoletus complex species [89,93] are overestimated in UV-light traps, although the Obsoletus complex species can be also underestimated [90]. Although a weak linear correlation has been found between UV-light and animal-baited abundances in horses of some *Culicoides* species [93], the interpretation of the relation between the abundances using both methods is not yet clearly established. Hence, our estimates of *m_ij_* might be overestimated, however, the models showed that more than 87% and 94% of outbreaks (BTV-1 and BTV-4, respectively) in Spain fell in areas with risk of transmission.

Due to the above-mentioned uncertainties, giving the threshold derived from the *R*_0_ definition of one, above which the disease would persist, would be arbitrary; and that is why using three categories to show the risk of BTV transmission for each month is more appropriate. Likewise, the external assessment of the monthly transmission risk maps proves this categorization convenient since the majority of the historically reported outbreaks fell into high and medium at-risk areas (Table 2). External assessment for the Balearic Islands was not possible since no accurate data of the outbreaks’ location was available. The low disagreement found in the external assessment can be partially explained because we are using predicted mean temperatures [50] and steady *Culicoides* spp. predicted abundance, without addressing annual variations in terms of climatic conditions. In addition, we did not include other *Culicoides* species that could be involved in BTV transmission in Spain, such as the Pulicaris complex [101], *Culicoides dewulfi* [102], *Culicoides chiopterus* [19], *Culicoides circumscriptus* and *Culicoides paolae* [20]. This could have also led to a slight overestimation of the transmission risk of areas inhabited by *C. imicola* in months of lesser abundance of this species (April-June) in comparison with Palearctic species, and therefore a slight underestimation of the risk of transmission in northern areas.

Another source of potential limitation is that we have deemed the host population as static and changeless through the months of study. Likewise, we have not included wildlife hosts in the model when red deer is potentially a BTV reservoir due to its long viremia [103]. However, in France, it was determined that red deer did not contribute to BTV maintenance [104], while in Spain the BTV wildlife cycle is more or less independent from the domestic, even more so with higher red deer abundance [105]. In addition, we have not fully addressed the *Culicoides* spp. feeding patterns, which are included in the study in the vector preference for host (*σ*). *Culicoides* spp. can feed in a wide variety of hosts depending on the species [106], including wild ruminant species for the *Culicoides* species that belong to the Obsoletus complex [107].

In our study, July is the month of maximal *R*_0_ transmission in Spain, followed by August, June and September. Our results are in agreement with Napp et al. [98] who studied the *R*_0_ during the epidemic wave of BTV-1 in the southern region of Andalusia. In their study, *R*_0_ reached its peak in July, and progressively decreased until almost reaching zero in November [98]. Our results are also comparable to the ones obtained in Austria, where the Obsoletus complex species are the major vector, in which higher *R*_0_ values were found between June-August [38]. In the case of strains that have been previously circulating during the previous year in areas where the Obsoletus complex species are more abundant, maximum vaccination coverage should be reached before June, since there is a quantum leap in the *R*_0_ between May and June. Although there is not a strong increase of *R*_0_ values between months when only considering *C. imicola*, vaccination campaigns should be ended before July.

For most years, October is the month of greater transmission except for BTV-1 during 2008–2010, when the virus was circulating in northern regions [26]. According to the cumulative outbreak data (Table 2), for both BTV strains there is a peak of notifications in October, although *R*_0_ is lower that month. Between May and June there is a noticeable leap in the mean *R*_0_ values, and according to our results in June-August the *R*_0_ is higher in Spain (Figure 6). Therefore, at the beginning of the period of greater estimated BTV transmission, few farms were infected, but they increased progressively through the months of greater risk of transmission. *R*_0_ shows the secondary cases derived from a primary case, and in October the number of cases is higher, showing that midge abundance and climatic conditions are still favorable for BTV transmission, in particular for areas where *C. imicola* is more abundant (Figure 5). Our study considered all susceptible host populations as naïve and showed at-risk areas without taking into account infected or recovered hosts, either naturally or artificially immunized. Although the model was not designed for an epidemic wave nor an endemic scenario, in which the same strain has been circulating several years, as had happened in Spain several times [7], it could be adapted into a susceptible-infected-recovered model. In addition, our model could be combined with atmospheric dispersion models specifically designed for *Culicoides* spp. [87], to simulate the arrival of infected midges from north Africa and simulate BTV spread thereafter, being able to take into account the seasonality of vector populations. However, even as it is, it is useful to highlight areas of higher transmission regardless of which strain/s might be circulating, and therefore can be useful to improve surveillance and design control and eradication programs more accurately.

In this study, we have identified a natural barrier that hampers the spread of BTV between northern and central-southern at-risk areas of BTV transmission in mainland Spain. This barrier occurs as a consequence of different temperatures in between both at-risk areas (Figure S1). These low mean temperatures prevent the distribution of *C. imicola* (Figure 2) and limit the distribution of the Obsoletus complex species during the majority of months (Figure 3). The lower *m_ij_*, along with the influence on temperature-dependent variables, significantly restrain BTV transmission in the *R*_0_ models for this area. Changes in climatic conditions —i.e., increase of the mean temperature— due to climate change may alter, and even erase this natural barrier. Future studies could be conducted to simulate possible modifications due to the increase in temperature. Only in June is this barrier less clear. According to both *R*_0_ (Figure 6) and the estimated date of infection of notified outbreaks (Table 2), BTV transmission is beginning to increase but is still lower during this month in comparison with the subsequent month. Therefore, it is unlikely that transmission can occur between these two well-defined risk areas. 

Historically, the majority of strains that have circulated in Spain were introduced through the south [7], and that is why the northern at-risk area has been less affected than the southern one. However, in November 2007, outbreaks of BTV-1 were reported in the northern province of the Basque Country [108], separated by hundreds of kilometers from the nearest BTV-1 reported outbreaks in central Spain [26]. After ruling out animal movement from BTV-1 affected areas, the possible long-distance wind-borne transportation of infected midges was pointed out as the possible means of introduction into the region [108]. The possibility of transmission due to short-range dispersal, which can reach a maximum of 5 km [109], of infected *Culicoides* spp. is very unlikely since it was a novel strain in Spain that produced clinical signs and it was not detected in between [108]. This event showed the implication of the Obsoletus complex species as competent vectors in Spain [8], as it is for northern Europe [17,18], and reinforces our results. However, it also raises the concern that strains of BTV-4 and other serotypes could circulate in northern Spanish areas as observed in countries further north [110]. More studies are needed to determine if the scarce abundance of *C. imicola* in northern areas of Spain could play a role in the management of the control and eradication of the disease.

## 5. Conclusions

In this study we have identified areas of greater risk of BTV transmission during April-October in Spain, with a formulation that enables the consideration of the dissimilarity in vector capacity of *C. imicola* and the Obsoletus complex species [31], although, more field or laboratory studies are required to better address those differences. We have also identified two large areas at risk of BTV circulation; the northern area, where the predominant vectors are the Obsoletus complex species, and the central-southern area, where *C. imicola* is more abundant. Both areas are separated by a natural barrier determined by temperature in which transmission is predicted to be low. Climate change could alter this barrier and/or its seasonality. The external validation performed shows that our models have correctly identified areas at risk of BTV transmission and that our results can certainly be of significance to better focus resources of BTV control and surveillance programs during the months of April-October in Spain. Our methodology can be extrapolated to other regions of the Mediterranean Basin where *C. imicola* and the Obsoletus complex species are major BTV vectors. To apply it to a larger scale, the harmonization of midge catches should be achieved so the standardization of abundances obtained with different trap types can be avoided [111]. In addition, the methodology described here could be applied to ecological studies and studies of arbovirus whose biological vector are insects of the *Culicoides* genus, namely African horse sickness virus, Schmallenberg virus and epizootic hemorrhagic disease virus, among others.

## Figures and Tables

**Table 1:** Parameters for *R*_0_ equations.

Non-Temperature Dependent Variables			
Variable	Variable Description	Selected Value (Range) or Formula	Reference
*b*	Probability of transmission from vector to host	0.9 (0.8–1.0)	[78]
*β_j_*	Probability of transmission from host to vector type *j*	0.02	[31,75,79]
*m_ij_*	Ratio of vectors (*N_j_*) to hosts (*H_i_*)	NjHi (0–5000)	[31]
*φ_Cj_*	Proportion of vectors type *j* attracted to cattle	$\varphi_{C}=\frac{m_{s}}{m_{s}{\;+\;\sigma m}_{c}}$	[31]
*φ_Sj_*	Proportion of vectors type *j* attracted to small ruminants	$\varphi_{S}=1\;-{\;\varphi }_{C}$	[31]
*σ*	Vector preference for host	0.15 (0–1)	[31,75,79]
*r_C_*	Recovery rate of cattle (1/duration of viremia)	0.0485	Duration of viremia (20.6) estimated by fitting a gamma distribution to data presented in [80], by [75]
*r_S_*	Recovery rate of sheep (1/duration of viremia)	0.0610	Duration of viremia (16.4) estimated by fitting a gamma distribution to data presented in [81] and [82], by [75]
*d_C_*	Mortality rate of cattle	0	[83]
*d_S_*	Mortality rate of sheep	0.0078 (0.001–0.01)	[79,83]
**Temperature (*θ*) dependent variables**			
**Variable**	**Variable Description**	**Formula**	**Reference**
*a*_1_	Biting rate for *C. imicola*	a(θ)=0.00014 θ (θ−3.6966) (41.8699 −θ)12.7056	[79]
*a*_2_	Biting rate for the Obsoletus complex species	a(θ)=0.000171 θ (θ−3.6966) (41.8699 − θ)12.7056	[84]
*μ_j_*	Natural mortality rate of vector type *j*	estimated by the Hermite cubic interpolation of temperature values of [85,86]	[87]
*v_j_*	Virogenesis rate in the vector type *j* (1/EIP)	v(θ)=0.0003 θ (θ − 10.4057)	[84]

**Table 2:** External assessment of the bluetongue virus (BTV) transmission risk maps using BTV-1 (2007–2017) and BTV-4 (2010–2018) historical data from the European Animal Disease Notification System (ADNS) database according to the estimated date of infection. The number and decimal fraction of the outbreaks that fall into the different risk categories is shown.

BTV-1							
Risk Category	April	May	June	July	August	September	October
1	1 (0.5)	0 (0)	5 (0.03)	33 (0.03)	78 (0.05)	458 (0.12)	448 (0.10)
2	1 (0.5)	3 (0.33)	129 (0.72)	125 (0.13)	552 (0.34)	2084 (0.56)	2455 (0.56)
3	0 (0)	6 (0.67)	44 (0.25)	833 (0.84)	1017 (0.62)	1147 (0.31)	1477 (0.34)
**Total number of outbreaks**	2	9	178	991	1647	3689	4380
**BTV-4**							
**Risk category**	**April**	**May**	**June**	**July**	**August**	**September**	**October**
1	0 (0)	0 (0)	-	-	0 (0)	8 (0.05)	15 (0.06)
2	0 (0)	0 (0)	-	-	2 (0.50)	70 (0.44)	157 (0.62)
3	1 (1)	1 (1)	-	-	2 (0.50)	80 (0.51)	83 (0.33)
**Total number of outbreaks**	1	1	0	0	4	158	255

**Table 3:** Statistical analysis of the difference between the observed BTV outbreaks and the number of expected BTV outbreaks calculated from the expected probability, which is based on the number of cells per each risk category.

BTV-1						
Month	Risk Category	Expected Probability	Observed Outbreaks	Expected Outbreaks	Residuals	Χ^2^ *
June	1	0.4	5	71.2	−7.85	112.02
	2	0.49	129	87.22	4.47	
	3	0.11	44	19.58	5.52	
July	1	0.45	33	445.95	−19.55	2740.5
	2	0.36	125	356.76	−12.27	
	3	0.19	833	188.29	46.98	
August	1	0.52	78	856.44	−26.6	3107.3
	2	0.33	552	543.51	0.36	
	3	0.15	1017	247.05	48.99	
September	1	0.57	458	2102.73	−35.87	3331.9
	2	0.32	2084	1180.48	26.3	
	3	0.11	1147	405.79	36.8	
October	1	0.65	448	2847	−44.96	7012.7
	2	0.27	2455	1182.6	37	
	3	0.08	1477	350.4	60.18	
**BTV-4**						
**Month**	**Risk category**	**Expected probability**	**Observed outbreaks**	**Expected outbreaks**	**Residuals**	Χ^2^ *
September	1	0.57	8	90.06	−8.65	307.86
	2	0.32	70	50.56	2.73	
	3	0.11	80	17.38	15.02	
October	1	0.65	15	165.75	−11.71	442.06
	2	0.27	157	68.85	10.62	
	3	0.08	83	20.4	13.86	

* df 2; *p* < 0.001.

## Supplementary Materials

The following are available online at https://www.mdpi.com/1999-4915/12/10/1158/s1, Table S1: Monthly numbers and percentages of *Culicoides* spp. positive catches (presence) in the entire dataset and in the occurrence models training dataset after the application of the Synthetic Minority Over-sampling Technique (SMOTE) algorithm, Table S2: *Culicoides* spp. occurrence and abundance models performance, Table S3: Monthly variable importance of the *Culicoides* spp. occurrence models through the mean decrease Gini (MDG), and abundance models through the increase in node purity (INP), Figure S1: WorldClim monthly mean temperatures for mainland Spain and the Balearic Islands. The climatic data presented here is available online: https://worldclim.org/. Administrative boundaries provided by Instituto Geográfico Nacional (ign.es); BDDAE CC-BY 4.0.
