# Supplementary material for: Identifying Spanish Areas at More Risk of Monthly BTV Transmission with a Basic Reproduction Number Approach
Source: Viruses. 2020 Oct 13;12(10):1158. doi: 10.3390/v12101158 (PMC7602074; doi:10.3390/v12101158)
Supplement: Supplementary file 1 [file viruses-12-01158-s001.pdf]

**Table S1.** Monthly numbers and percentages of *Culicoides* spp. positive catches (presence) in the entire dataset and in the occurrence, models training dataset after the application of the Synthetic Minority Over-sampling Technique (SMOTE) algorithm.

| ***Culicoides* species** | **Month** | ***Culicoides* spp. catches in the entire dataset** | | ***Culicoides* spp. catches in the training dataset after SMOTE** | |
|---|---|---|---|---|---|
| | | **Positive catches/Total** | **% (SE)** | **Positive catches/Total** | **% (SE)** |
| ***C. imicola*** | April | 72/331 | 0.22 (0.02) | 104/260 | 0.40 (0.03) |
| | May | 85/331 | 0.26 (0.02) | 120/300 | 0.40 (0.03) |
| | June | 91/331 | 0.27 (0.02) | 134/335 | 0.40 (0.03) |
| | July | 114/331 | 0.34 (0.03) | 120/268 | 0.45 (0.03) |
| | August | 126/331 | 0.38 (0.03) | 127/271 | 0.47 (0.03) |
| | Septem ber | 128/331 | 0.39 (0.03) | 135/270 | 0.50 (0.03) |
| | October | 121/331 | 0.37 (0.03) | 126/273 | 0.46 (0.03) |
| **Obsoletus complex** | April | 189/331 | 0.57 (0.03) | 127/231* | 0.55 (0.03) |
| | May | 232/331 | 0.70 (0.03) | 149/291 | 0.51 (0.03) |
| | June | 232/331 | 0.70 (0.03) | 149/291 | 0.61 (0.03) |
| | July | 241/331 | 0.73 (0.02) | 144/270 | 0.53 (0.03) |
| | August | 206/331 | 0.62 (0.03) | 141/274 | 0.51 (0.03) |
| | Septem ber | 198/331 | 0.60 (0.03) | 144/265 | 0.54 (0.03) |
| | October | 181/331 | 0.55 (0.03) | 123/231* | 0.53 (0.03) |

*The SMOTE algorithm was not applied.

**Table S2.** *Culicoides* spp. occurrence and abundance models performance.

| *Culicoides* species | Month | Occurrence models | | | | | Abundance models | |
|---|---|---|---|---|---|---|---|---|
| | | Precision | Sensitivity | Specificity | F1 | AUC[a] | MAE[b] | RMSE[c] |
| ***C. imicola*** | April | 0.7 | 0.7 | 0.92 | 0.7 | 0.81 | 0.31 | 0.49 |
| | May | 0.78 | 0.88 | 0.92 | 0.82 | 0.9 | 0.45 | 0.62 |
| | June | 0.59 | 0.83 | 0.82 | 0.69 | 0.82 | 0.53 | 0.78 |
| | July | 0.7 | 0.82 | 0.82 | 0.76 | 0.82 | 0.55 | 0.79 |
| | August | 0.8 | 0.85 | 0.85 | 0.82 | 0.85 | 0.58 | 0.77 |
| | September | 0.78 | 0.84 | 0.85 | 0.81 | 0.85 | 0.66 | 0.88 |
| | October | 0.7 | 0.76 | 0.81 | 0.73 | 0.78 | 0.61 | 0.88 |
| | Average | 0.72 | 0.81 | 0.86 | 0.76 | 0.83 | 0.53 | 0.74 |
| **Obsoletus complex** | April | 0.75 | 0.68 | 0.63 | 0.71 | 0.65 | 0.59 | 0.72 |
| | May | 0.85 | 0.64 | 0.71 | 0.73 | 0.68 | 0.66 | 0.77 |
| | June | 0.82 | 0.75 | 0.57 | 0.78 | 0.66 | 0.62 | 0.79 |
| | July | 0.85 | 0.73 | 0.67 | 0.79 | 0.7 | 0.71 | 0.88 |
| | August | 0.76 | 0.67 | 0.67 | 0.71 | 0.67 | 0.7 | 0.89 |
| | September | 0.64 | 0.75 | 0.57 | 0.69 | 0.66 | 0.54 | 0.67 |
| | October | 0.77 | 0.71 | 0.71 | 0.74 | 0.71 | 0.58 | 0.71 |
| | Average | 0.78 | 0.70 | 0.65 | 0.74 | 0.68 | 0.63 | 0.78 |

[a]AUC: area under the receiver operating characteristic curve
[b]MAE: mean absolute error
[c]RMSE: root mean squared error.

**Table S3.** Monthly variable importance of the *Culicoides* spp. occurrence models through the mean decrease Gini (MDG), and abundance models through the increase in node purity (INP).

| | | | | 1 | 2 | 3 | 4 | 5 |
|---|---|---|---|---|---|---|---|---|
| **Occurrence models** | *C. imicola* | April | Variable | LSTn | ALT | PREC | LSTd | LD |
| | | | MDG | 29.41 | 13.77 | 11.39 | 10.8 | 10.03 |
| | | May | Variable | LSTn | PREC | LSTd | LD | OCTOP |
| | | | MDG | 35.28 | 25.99 | 11.51 | 10.72 | 6.1 |
| | | June | Variable | PREC | LSTn | LD | LSTd | ALT |
| | | | MDG | 53.92 | 24.58 | 15.4 | 8.18 | 7.89 |
| | | July | Variable | PREC | LSTn | LSTd | ALT | OCTOP |
| | | | MDG | 23.54 | 18.06 | 10.69 | 8.43 | 7.64 |
| | | August | Variable | LSTn | PREC | OCTOP | LD | LSTd |
| | | | MDG | 31.59 | 23.18 | 11.56 | 9.92 | 7.96 |
| | | September | Variable | LSTn | PREC | LSTd | SILT | OCTOP |
| | | | MDG | 44.08 | 23.59 | 17.63 | 6.43 | 5.43 |
| | | October | Variable | LSTd | LSTn | ALT | LD | PREC |
| | | | MDG | 37.12 | 19.34 | 10.26 | 8.76 | 8.14 |
| | **Obsoletus complex** | April | Variable | ALT | NDVI | LSTd | Silt | MIR |
| | | | MDG | 11.82 | 8.38 | 8.12 | 7.89 | 7.7 |
| | | May | Variable | LSTd | MIR | ALT | NDVI | EVI |
| | | | MDG | 9.55 | 9.24 | 9.12 | 9.02 | 8.87 |
| | | June | Variable | PREC | LSTd | LSTn | LD | NDVI |
| | | | MDG | 12.67 | 12.41 | 11.34 | 8.6 | 7.87 |
| | | July | Variable | PREC | LSTd | MIR | NDVI | Silt |
| | | | MDG | 14.99 | 9.37 | 8.95 | 8.23 | 7.84 |
| | | August | Variable | LSTn | PREC | EVI | LD | ALT |
| | | | MDG | 17.71 | 8.89 | 7.95 | 7.86 | 7.77 |
| | | September | Variable | OCTOP | LSTd | PREC | LSTn | Sand |
| | | | MDG | 11.19 | 11.09 | 8.69 | 8.68 | 8.51 |
| | | October | Variable | LSTd | Clay | Sand | Silt | PREC |
| | | | MDG | 11.65 | 9.91 | 9.77 | 8.93 | 8.26 |
| **Abundance models** | *C. imicola* | April | Variable | PO_IMIC | LSTn | LD | LSTd | ALT |
| | | | INP | 19.91 | 6.06 | 3.86 | 3.51 | 3.39 |
| | | May | Variable | PO_IMIC | LSTn | PREC | WS | LD |
| | | | INP | 28.81 | 8.69 | 5.95 | 4.26 | 4.18 |
| | | June | Variable | PO_IMIC | PREC | WS | LSTn | LD |
| | | | INP | 32.41 | 14.81 | 10.65 | 8.03 | 6.93 |
| | | July | Variable | PO_IMIC | PREC | LSTd | LSTn | OCTOP |
| | | | INP | 45.77 | 18.2 | 10.9 | 9.84 | 7.46 |
| | | August | Variable | PO_IMIC | PREC | LSTn | LSTd | LD |
| | | | INP | 38.68 | 27.43 | 19.35 | 9.77 | 9.2 |
| | | September | Variable | PO_IMIC | PREC | LSTn | LSTd | ALT |
| | | | INP | 30.73 | 24.9 | 19.54 | 10.21 | 10.06 |
| | | October | Variable | PO_IMIC | LD | LSTd | LSTn | WS |
| | | | INP | 58.93 | 10.84 | 7.02 | 6.98 | 5.79 |
| | **Obsoletus complex** | April | Variable | PO_OBSOL | MIR | LSTd | NDVI | PREC |
| | | | INP | 22.93 | 15.09 | 10.42 | 7.31 | 4.75 |
| | | May | Variable | PO_OBSOL | PREC | NDVI | ALT | SILT |
| | | | INP | 20.65 | 10.02 | 9.8 | 9.36 | 8.91 |
| | | June | Variable | PO_OBSOL | LSTd | NDVI | PREC | WS |
| | | | INP | 37.44 | 15.33 | 14.81 | 11.78 | 9.45 |
| | | July | Variable | PO_OBSOL | LSTd | PREC | MIR | LSTn |
| | | | INP | 21.26 | 19.47 | 16.48 | 9.85 | 9.23 |
| | | August | Variable | LSTd | PO_OBSOL | PREC | MIR | NDVI |
| | | | INP | 20.53 | 16.4 | 14.17 | 11.15 | 9.14 |

| | | | | | | |
|---|---|---|---|---|---|---|
| September | Variable | PO_OBSOL | LSTd | PREC | NDVI | MIR |
| | INP | 17.21 | 12.03 | 8.8 | 5.76 | 5.47 |
| October | Variable | PO_OBSOL | LSTd | EVI | NDVI | MIR |
| | INP | 11.58 | 8.22 | 7.76 | 7.65 | 6.31 |

NDVI: Mean Normalized Vegetation Index

EVI: Mean Enhanced Vegetation Index

MIR: Mean medium-infrared reflectance

LSTd: Mean day-time surface temperature

LSTn: Mean night-time surface temperature

PREC: Mean precipitation

WS: Mean wind speed

ALT: altitude

LD: Livestock density (sheep, cattle and goat)

OCTOP: Topsoil organic carbon content

PO_IMIC: Probability of occurrence of *Culicoides imicola*

PO_OBSOL: Probability of occurrence of the Obsoletus complex

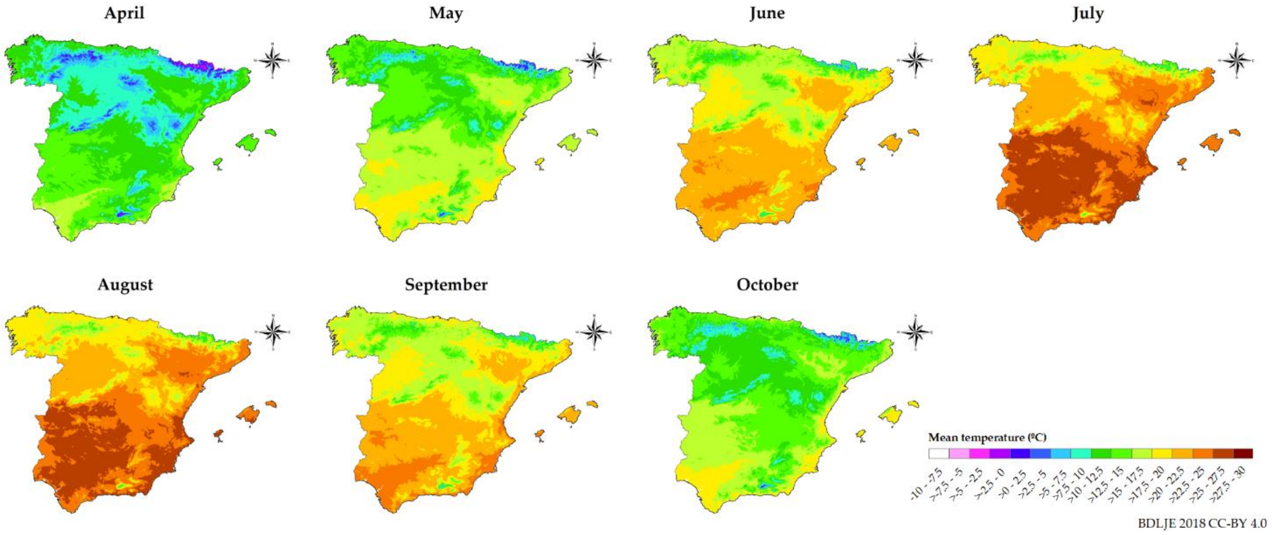


**Figure S1.** WorldClim [50] monthly mean temperatures for mainland Spain and the Balearic Islands. The climatic data presented here is available online: https://worldclim.org/. Administrative boundaries provided by Instituto Geográfico Nacional (ign.es); BDDAE CC-BY 4.0.
